# Correction: Self-referential encoding of source information in recollection memory

**DOI:** 10.1371/journal.pone.0271143

**Published:** 2022-07-05

**Authors:** Ross Lawrence, Xiaoqian J. Chai

The following information is missing from the Funding statement: X.C. is supported by the Natural Sciences and Engineering Research Council of Canada (RGPIN-2020-05520), Canada First Research Excellence Fund, awarded to McGill University for the Healthy Brains for Healthy Lives initiative, and the Canada Research Chairs program.
