# Supplementary material for: Self-referential encoding of source information in recollection memory
Source: PLoS One. 2021 Apr 15;16(4):e0248044. doi: 10.1371/journal.pone.0248044 (PMC8049320; doi:10.1371/journal.pone.0248044)
Supplement: S1 Text — Provides the steps and script used while conducting the experiment. (DOCX) [file pone.0248044.s001.docx]

**Required Material:**

Laptop/s

Presentation dongle

K-Bit IQ form plus booklet

Toy rewards

Young scientist certification

Consent form

Payment form

Family Questionnaire

**STUDY PROCEDURE**:

Any steps that are for child participants only will be in **Bold**, it is not necessary for adult participants to perform these steps.

1. Greet participant and explain what the study entails:
   1. *This study consists of three activities, which will be done one after the other with small breaks in-between to use the bathroom or get some water. The first activity involves practicing the task that we will need you to do inside of the MRI today. I’ll be explaining to you what exactly the task entails, and you will be practicing on a computer first, then we will:*
      1. [IF CHILD: *… practice with our dummy MRI machine to give an idea of what the real thing will be like and see if you are okay with continuing. The dummy MRI is made from an old MRI, with the magnet and other internal mechanisms removed. It won’t be able to take pictures of your brain, like the real MRI, but it will let us practice as much as we need. Once you are comfortable with the task and decide to continue, we will head over to the MRI room and get started.*]
      2. [IF ADULT: *… head over to the MRI observation room and get started with the study*.]
   2. *Once we head over to the MRI observation room, we’ll go over a checklist and sign a form to make sure that you are safe to enter the MRI machine. The MRI will take approximately 45 minutes, half of which you will spend watching a movie of your choosing. The second half of the MRI time will be either looking at a blank screen with a “+” on it or spent preforming the tasks we’ve practiced. I’ll be talking with you the whole time and you will be given an emergency button to use if you ever feel like you must take a break or stop the task.* [Mention that anyone with the participant is welcome to follow the participants and researchers and ask any questions they have]
   3. *Once the MRI is done, we’ll take you out of the machine and show you images of your brain. You can ask us any questions you have and take that time to go to the bathroom or get some water. You’ll then perform something called a K-Bit test with* [researcher] *which will take about 30 minutes. The test consists of some puzzles and trivia questions and doesn’t involve any computers or MRI machines.*
   4. *Once that is done you’ll perform a third activity for us, which I can’t specify as it is meant to be a secret. It will be done on a computer outside of the MRI machine and take about 20-30 minutes, depending on how quickly you complete it. Once that is complete, there will be another form to fill out about your payment, but after that you are all done and are free to go.* [IF CHILD: Tell guardian that you can tell them what the third activity is, as long as they do not inform the participant of it beforehand]
   5. *Any questions?*
2. Have subject or subject’s guardian read and fill out the consent form, answering any questions they may have.
3. **Take subject to practice MRI**
   1. Show participant practice MRI and answer any immediate questions
   2. *We are going to use this old MRI machine to practice what you will be doing for this study. An MRI machine allows us to take a bunch of pictures of your brain so we can look at them like a movie and see how it works. We are trying to figure out how children’s brains respond and remember things that they see. Just like taking a picture on a camera, you shouldn’t feel anything while you are inside.*
   3. *You’ve taken pictures before where something moves and the picture is all blurry. Well, in order to take good pictures, we need you to try to stay as still as possible while inside of the machine. Now, don’t think that you have to be stiff and tense up, just find a cozy position on your back and try not to move your head.*
   4. *The MRI machine is going to be loud when it is taking pictures of your brain, so in our practice MRI we have speakers to help simulate what it is going to be like. You will have headphones when inside of the MRI to help protect your ears, but it will be still be loud.*
   5. Show participant the buttons they will be using and answer any questions
4. Practice encoding exercise:
   1. Using the desktop attached to the practice MRI in the observation room, run the practice presentation files for the encoding exercise.
      1. *Now we are going to practice the activity that we need you to do while inside of the MRI. When you are inside the machine, there is going to be a little screen where we are going to show you pictures and ask you a question. We are going to give you two buttons, one for each hand. and all you need to do is press the button that matches with your answer for each picture.*
      2. Run the practice MRI Presentation file, holding on the first image (self question)
      3. *For each picture, you are going to see an object, like* [shown object] *on a background. Below you are going to see some symbols. When you see a smiling face and a neutral/bored face, we are asking you if you like the* [object] *or if you don’t care about/don’t like it. I like* [object]*, so I will press the button on left; if I didn’t care about it or didn’t like it, I would press the button on right.*
   2. Move to next image (animacy question)
      1. *See the new object and see the new symbols below the picture? When you see a leaf and a leaf with a red X over it, it is asking if you think* [object] *is alive. I think that* [object] *is alive, so I will press the button on the left side. If I didn’t think it was alive I would press the button on the right.*
   3. Have the participant practice pressing buttons on the computer using the practice encoding program.
   4. **Put the participant into the practice MRI, making sure to hand them the buttons**
      1. Ask them if they are okay/comfortable
      2. Reiterate that they need to try and stay still. *Remember, even though this is practice, we need you to try and keep your head as still as possible. What’s the most important thing? Keeping your head still.*
      3. Ask them to press each of the buttons to check if the practice MRI is registering them.
   5. **Begin the practice MRI**
      1. *Okay, we are going to start the activity. Remember, press the button on the side of your answer.*
      2. Turn on the recorded MRI sounds and run the practice Presentation file. Give feedback where applicable, if a participant is unable to complete the practice activity, end the study here.
5. Take subject to MRI observation room

[BATHROOM BREAK or WATER BREAK]

1. Explain the scanning procedure:
   1. **Voluntary:** *It may be difficult to stay still inside of the MRI, but we need you to do your best to stay still (but relaxed) and focused. It is going to take more time than the practice we just did, but you get to watch a movie for a half of that time. And if you ever need to get out of the machine, you will have a button you can squeeze and we will get you out. Don’t press that button if you are bored or if your nose itches, it is for emergencies. If you press that button I will think that you are saying “I have a problem and I need help NOW” and I will stop everything and talk to you.*
   2. **Metal:** *Make sure you have no metal on you, like belts, necklaces, earrings, hair clips, retainers, jewelry, etc.* Ask parents to do the same just in case they have to enter the MRI room to comfort their child during testing. [See MRI assent form for list of conditions that can prohibit entrance]
   3. **Noise:** *The machine is going to make a lot of funny and loud noises while you are inside of it. That’s why we’re giving you special headphones to block out the noise and let you talk to us. I’ll be talking to you between scans to make sure that you are doing ok. Between scans will be quieter, so I’ll be able to hear what you say on the microphones inside the MRI.*
   4. **Movement:** *While inside the machine, we need you to stay as still as you can. Don’t tense up and try too hard to stay still, try finding a comfortable position and relax.*
2. Have participant or participant’s guardian sign the assent form required to enter the MRI room. Participant can now select which movie they would like to watch.
   1. *Okay, now is your last opportunity to use the bathroom or get some water. You will be in the MRI for approximately 45 minutes and if we take you out of the MRI we will have to redo some of the scans, so we don’t want you to be taking a bathroom break halfway through.*

[BATHROOM BREAK or WATER BREAK]

1. Take participant into the MRI room and explain how to operate the two buttons required for the activity. Adjust the mirror and screen so that they can see the entirety of the projector screen.
   1. Before first scan:
      1. Check that they can hear you through their headphones
      2. Check that the buttons the participant has work properly
      3. *Ok, we are going to start with the first scan, it will take about 1 minute and will be somewhat loud.*
   2. After Survey Scan:
      1. *That was the first scan. How are you doing in there?*
      2. *The next scan will take about 5 minutes and I’ll be starting the movie now.*
      3. Start the movie
   3. After Structural Scan:
      1. *Great job, how are you doing?*
      2. *Next is a scan that will take 10-15 minutes, keep doing a good job and remember to try to stay as still as possible.*
   4. After Spectroscopy Scan:
      1. *You’re doing great! How are you feeling?*
      2. *Next is a scan that will take about 5 minutes, but we’ll have to turn the movie off for it. You are going to see a plus sign on the screen and all you need to do is keep your eyes open and look at the symbol. You can blink normally; we just need you to stay awake and focused.*
      3. Stop the move and switch projector to the fixation image
   5. After Resting Scan:
      1. *Great, you made it through the most boring part of the study! Now we are going to start the activity we practiced. There will be 4 scans of this activity, each about 5 minutes long, with new images each time. I’ll be checking in with you between scans. Do you remember how to do the activity?* [Reiterate instructions if necessary]
   6. After Encoding Activity Scans:
      1. *Great job! I’m coming in now to get you out of the MRI machine.*
      2. Remove participant from the MRI machine and show them images of their MRI scans. Offer water and a bathroom break before starting the K-Bit II cognitive test.

[BATHROOM BREAK]

1. Cognitive test
   1. Either take them to the waiting area attached to the MR2 observation room or the medical room by the Practice Scanner.
   2. Administer the K-Bit test. If the test takes longer than 30 minutes stop the test at the end of whatever module you are on and complete it after the memory test. [See K-Bit manual for instructions on how to administer the test]
2. Memory Test
   1. Load the practice memory test Presentation file
   2. *Okay, now we are going to see how well you remember the images shown to you inside of the MRI by giving you a test. You are going to be shown a series of objects and you have to decide if you saw the object while you were inside the MRI. We’ll also ask a few other questions about the images you saw. We have a short practice test to go over how to complete the activity.*
   3. Display first object
   4. *For each of the objects you see, you need to decide:*
      1. *If you are able to remember something specific about seeing the picture while you were in the scanner, for example, what button you pressed, what you were thinking about when the image was presented, or what the image looked like on the screen, etc, press the button for "****Remember****".*
      2. *If it is a new object that you haven’t seen before in the MRI, press the button for “****New****”****.*** *We have mixed in new pictures to test your memory.*
      3. *If you recognize that the picture was shown to you inside of the MRI but you can't recollect anything specific about experiencing the item, press the button for "****Familiar****".*
   5. *We are only asking about the objects or images you saw while doing the activities we practiced. If you saw something in the movie you watched, but not the MRI activity, do not answer Remember or Familiar*
   6. Press a button to move it to the next question
   7. *Now you have to decide what background was shown with the object. If you remember the object in front of the beach, press this button, if you remember it in front of the forest, press this button.*
   8. Press a button to move it to the next question
   9. *Now you have to decide what question you were asked with the object. If you remember being asked if you liked the object, press this button, if you remember being asked if it was alive, press this button. When you do that you will move on to the next object.*
   10. Complete practice test with the participant and answer any questions/correct any erroneous answering methods. Load the memory test Presentation file and have the participant begin.
   11. *Please take as much time as you need to make the most accurate decision. Don’t worry about making a mistake, we want you to be relaxed and doing your best.*
3. Post study forms and questions
   1. *Great job! Now that you are done, I can answer any questions you have about the study.*
   2. Answer any further questions from participant or participant’s guardian
   3. Complete payment form
   4. Complete post-study survey
   5. [IF CHILD: Allow child to select 2 toys from the toy box]
