# Supplementary material for: Self-referential encoding of source information in recollection memory
Source: PLoS One. 2021 Apr 15;16(4):e0248044. doi: 10.1371/journal.pone.0248044 (PMC8049320; doi:10.1371/journal.pone.0248044)
Supplement: S2 Text — Explains the labeling structure used for the MultiTree models used in the MPT analysis. (DOCX) [file pone.0248044.s002.docx]

**­MPT Model Assumptions and Constraints:**

A key assumption was made during the creation of the MPT model, namely that all participants were truthful in their initial judgement of recognition/answering as New. No participant did not recognize the image presented before them but still chose to answer either Remember/Familiar. This assumption was made to resolve the parameters present in the model, with the idea that the percentage of total answers in which the participant lied was not statistically significant. Due to how granular the answers of each participant were divided in the MPT model, there were limitations on the data collected for a few participants where the number of certain responses was 0 (i.e. answering both source questions correctly for an item which they said that was Familiar and was presented during the encoding phase in front of a garden background with the self-encoding prompt). As the MPT model requires non-zero values, all recorded values were multiplied by 1000 and the zero values were given a value of one, which allows for the calculation of the model parameters while not being statistically significant.

**MPT Model Parameter Labelling Guide:**

Each MPT model is broken into 5 trees, 4 trees separated by the combination of background (G = garden or B = beach) and encoding prompt (S = self-referential, L = semantic) with a fifth tree for the newly seen images during the testing activity (New).

Two versions of the MPT model were made, with the same 5 trees but with a different order of parameters. To analyse the dependence of the two pieces of source information on each other, the order by which the “splits” in the model occur are changed. This results in the Background-Prompt model calculating the probability that the participant properly remembered the encoding question dependent on their answer for the background, while the Prompt-Background model calculates the probability that the participant properly remembered the background dependent on their answer for the encoding prompt.

In the description below, the term “Split” references the binary branching steps from left to right. For example, in Figure 1, Split 1 entails pAGS and (1-pAGS), Split 2 entails pR1 and (1-pR1), Split 3 entails pRG1, (1-pRG1), pFG1 and (1-pFG1), etc. For the following explanations of what each split represents, which are generalized to apply to all non-New trees, let # be a placeholder for any number 1-4 and let ? be a placeholder for a letter.

**Background-Prompt Model (Figure 1):**

**Split 1:** The probability that the participant recognizes the image (pA??) or incorrectly says that it is New, leading to the Miss_?? constant (see below).

**Split 2:** The probability that the participant says they Remember the image (pR#) or that the image is Familiar (1-pR#).

**Split 3:** The probability that the participant says that the image was shown over the garden background (p?G?#) or the beach background (1-p?G?#)

**Split 4:** The probability that the participant says that the image was displayed with the self-referential encoding prompt (p??S#) or the semantic encoding prompt (1-p??S#)

**Prompt-Background Model (Figure 2):**

**Split 1:** The probability that the participant recognizes the image (pA??) or incorrectly says that it is New, leading to the Miss_?? Constant (see below)

**Split 2:** The probability that the participant says they Remember the image (pR#) or that the image is Familiar (1-pR#).

**Split 3:** The probability that the participant says that the image was displayed with the self-referential encoding prompt (p?S?#) or the semantic encoding prompt (1-p?S?#).

**Split 4:** The probability that the participant says that the image was displayed over the garden background (p??G#) or the beach background (1-p??G#)

**New Item Tree (Figure 3):**

An important distinction needs to be made between the 4 trees described in both the Background-Prompt and the Prompt-Background MultiTree models (i.e. the trees related to items seen during the encoding question) and the “New” tree for each model. In each of the models exists a 5^th^ tree which follows the same split pattern described above, but with the key distinction of pAN and (1-pAN) in the first split denoting the probability that the image was correctly deemed New (pAN) or incorrectly deemed to have appeared in the encoding task (1-pAN). In the proceeding splits, the letter “b” (meaning *bias*) replaces the letter “p” as a way to clearly distinguish that these probabilities are attempting to measure the bias in answering patterns of participants when they erroneously say they recognize an item. Figure 3 is an example of the New tree for the Background-Prompt model.

**Tree Origin Points:**

G_Self : Images presented over the garden background with the self-referential encoding prompt.

G_Live : Images presented over the garden background with the semantic encoding prompt

B_Self : Images presented over the beach background with the self-referential encoding prompt.

B_Live : Images presented over the beach background with the semantic encoding prompt.

New : Images not shown to the participant during the encoding activity.

**Tree Constants Key:**

The parameters and constants in each of the trees use various abbreviations, listed below. The right-most “boxes” in each tree represent the recorded amount of each answer for a given participant. For example, in Figure 1,

R : Remember

F : Familiar

G : Garden

B : Beach

S : Self-referential

L : Semantic

1 : Image displayed with garden background and self-referential encoding question

2 : Image displayed with garden background and semantic encoding question

3 : Image displayed with beach background and self-referential encoding question

4 : Image displayed with beach background and semantic encoding question

Miss_ : Incorrectly answering that a previously seen image during the encoding activity was New.

All_ : Number of images that the participant correctly recollected both the image and both pieces of source information.

FA_ : False Alarm, or items not shown during the encoding task that the participant erroneously answered that they had recognized.

-Q : Number of images that the participant correctly recollected, got the background correct, but did not get the encoding prompt correct.

-B : Number of images that the participant correctly recollected, got the encoding prompt correct, but did not get the background correct.

-BQ : Number of images that the participant correctly recollected, but failed to correctly recollect both pieces of source information.

**Figures:**


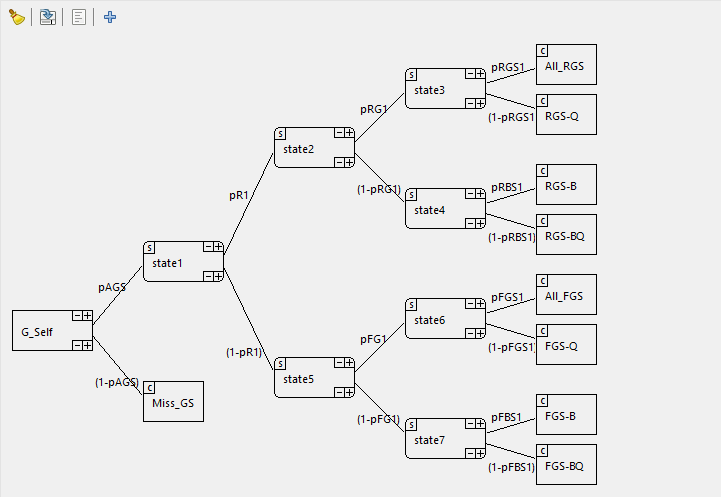


**Figure 1:** Background-Prompt MultiTree model tree for items displayed with the garden background and self-referential encoding prompt


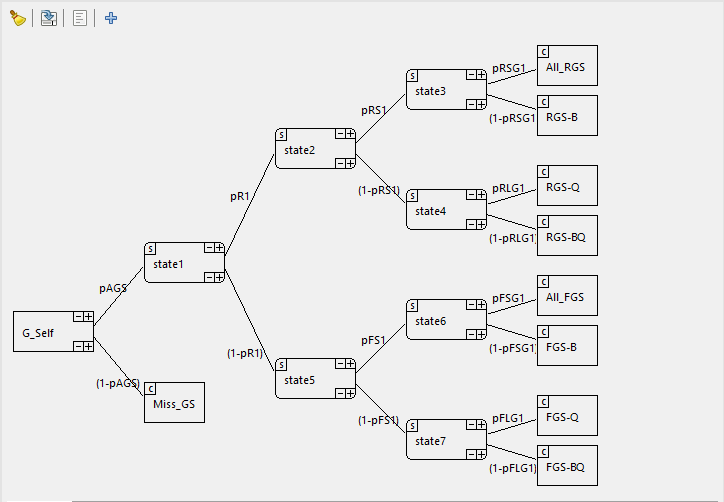


**Figure 2:** Prompt-Background MultiTree model tree for items displayed with the garden background and self-referential encoding prompt


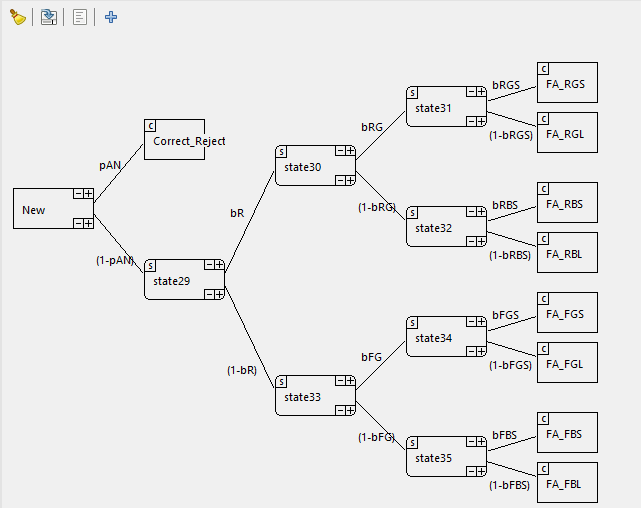


**Figure 3:** Background-Prompt MultiTree model tree for new items not seen during the encoding task. Note the use of the letter “b” instead of “p” to denote that this parameter represents a bias response, or a response made by the participant about information they could not know.
