# Supplementary material for: Self-referential encoding of source information in recollection memory
Source: PLoS One. 2021 Apr 15;16(4):e0248044. doi: 10.1371/journal.pone.0248044 (PMC8049320; doi:10.1371/journal.pone.0248044)
Supplement: Attachment — Submitted filename: Response to Reviewers.docx [file pone.0248044.s003.docx]

**Manuscript PONE-D-20-04302**

**Response to Reviewers**

Dear Dr. Barbara Dritschel,

We would like to thank you and both reviewers for noting the strengths of the manuscript, and for their helpful and constructive comments. We have addressed each of the comments raised by the reviewers in the revised manuscript, and reanalyzed the data using the IRK method suggested by both reviewers. Below is our point-by-point response. Responses given by reviewers that were actionable are colored for clarity, with author responses interspersed within.

Sincerely,

Ross Lawrence, Xiaoqian Chai

**Reviewers’ Comments to the Authors:**

**Reviewer #1:**

Reviewer #1: There are both strengths and weaknesses in this paper. It is an interesting research question that may help explain some autobiographical memory (assuming that much of our autobiographical memory is based on self-referential processing). However, I don’t think the paper should be published in its current form. I note the reasons for my opinion below, in the spirit of improving the research.

The most important issue is that R and K (or F) responses cannot be entered into a single ANOVA with response type as a factor. R and K responses are mutually exclusive, so they are not independent from each other and therefore violate the assumptions of ANOVA. The only way they can be compared to each other is by using the IRK (independent RK) method described by Yonelinas & Jacoby, 1995 (The relations between remembering and knowing as bases for recognition: Effects of size congruency). This creates a probability estimate of familiarity. As a result of the way the analyses were done, it is difficult to review the conclusions because it’s possible that the results will change. Regardless of the publication decision here, these data have to be reanalyzed before submitting again.

Author Response: We thank the reviewer for nothing the strength of our study for raising this important point on estimating familiarity. We have re-analyzed our data using the independent RK method from Yonelinas & Jacoby 1995. Familiarity rate was re-estimated to take into account of the proportions of Remember trials. We found similar results as before on the effect of self-referential encoding on memory recollection. Self-encoding resulted in significantly higher memory accuracy for R trials but not for K trials (Page 12-13).

Pleasantness judgments are typically thought of as deep processing, rather than being self-referential. Is there evidence about this? That is, I was under the impression that self-referential processing produced better memory than pleasantness judgments.

Author Response: We modeled the “like/do not like” paradigm after Cunningham et al. 2014, Gusnard et al, 2001, Leshikar & Duarte 2012, to assess self-related process of information. The choice was made with the consideration of feasibility with children in mind, as this dataset was part of a larger fMRI study that included children (ages 7 and above). Compared to some of the other self-referential paradigms (e.g., judging whether a word describes self), this task was easy to explain and test in children, and elicits subjective judgement based on personal preferences. We agree with the reviewer that other self-referential paradigms may have a more pronounced facilitation effect on memory. Nevertheless, with this task we still saw robust memory enhancement in the self-encoding condition compared to semantic encoding.

*References:*

Cunningham, S.J., Brebner, J.L., Quinn, F. and Turk, D.J. (2014), The Self‐Reference Effect on Memory in Early Childhood. Child Dev, 2014, 85: 808-823. Doi:10.1111/cdev.12144

Gusnard, D. A., Akbudak, E., Shulman, G. L., Raichle, M. E. Medial prefrontal cortex and self-referential mental activity: Relation to a default mode of brain function. Proceedings of the National Academy of Sciences Mar 2001, 98 (7) 4259-4264; DOI: 10.1073/pnas.071043098

Leshikar, E. D., and Duarte, A. Medial prefrontal cortex supports source memory accuracy for self-referenced items. Social neuroscience. 2012; *7*(2), 126–145. Doi:10.1080/17470919.2011.585242

Many people who use the RK procedure actually use and advocate for the RKG (guess) method. Was there a specific reason to exclude the guess response?

Author Response: We agree with the reviewer the RKG method may lead to more accurate identification of the different memory categories (familiar and recollection), by separating out the guesses. We kept the traditional RK procedure to compare with prior studies on self-referential effect of recollection. In addition, as mentioned above, to make the task work in children, we decided not to include the guess option.

The RK instructions need to be described in a lot of detail, given that brief instructions without practice can lead to a confusion of how to use the responses. A copy of the instructions would be appropriate (perhaps in an appendix if they are long). This will be important for further review of the manuscript.

Author Response: All participants were given a practice after the instruction, before beginning the task. We have included a copy of the instructions in the supplemental material. Participants were instructed to make the “Remember” judgement if they remember seeing the object during the study session and can recall additional information of the episode such as what they were thinking at the time, and make the “Familiar” judgement if they think they have seen the object in the study phase but were unable to remember anything specific.

Having a source question after an F judgment implies to subjects that they should/could have source available. By definition of the procedure though, remember responses include details and know responses do not. If there is recollected detail in a know response, it’s arguable that the procedure is just not being followed correctly. This has implications for whether or not we can conclude that subjects had some accurate source memory (and that it differed between conditions) for familiarity-based memory. This should be discussed.

Author Response: We agree with the reviewer that K responses are less likely to contain specific details of the encoding episode and the source responses may largely contain guesses. For this reason and the fact that there are very few trials of the K responses, we have decided to restrict the source accuracy analysis to only the “Remember” responses.

p should be reported as p = .xxx, unless it’s less than .001, which can be reported as p < .001.

Author Response: We have made the change in the revised manuscript.

**Reviewer #2:**

First and foremost, I wish the authors well in this unprecedented time and hope that they and their families remain safe and healthy.

This paper presents a single experiment examining the effect of self-referential encoding on item and source memory. Participants incidentally encoded objects overlaid on a scene background and were prompted to make a self-referential (pleasantness) or semantic (living/nonliving) judgment for each trial. At test, participants first made a Remember/Familiar/New judgment for old and new objects. Objects attracting a R or F responses were further probed for source memory of the peripheral detail (background scene) and central detail (self vs. semantic). Self-referential encoding increased the accuracy of Remember responses and decreased the accuracy of Familiar responses. Self-referential effects were observed for the peripheral and central details, which was particularly evident for Remember trials. These findings are taken as evidence that self-referential encoding creates a more elaborate and rich memory trace that supports recollection for many different features of an event.

The Introduction does a good job outlining the gaps in the literature about the effects of self-referential encoding on source memory. The experiment was also well designed and to my read there are no major flaws in the design that would confound interpretation of the results. I particularly like the inclusion of peripheral versus central source details in the design, which does help expand research on this topic. I do have some concerns regarding the analysis, particularly the calculation of memory measures, and interpretation of the results that I express below, along with some minor suggestions.

Major Points:

1) The proportion of Remember and Familiar responses are directly compared in the item memory analysis, which is problematic because these proportions are not independent of one another. A higher proportion of R responses reduces the highest proportion of F responses that can be observed. With this analysis approach, it is difficult to determine if different encoding tasks are leading to the trade off or if the difference is simply a statistical artifact. To be clear, this criticism does not apply to the self-referential encoding advantage on the R Hit-FA score reported in the paper, but mainly to the F accuracy measure. I would recommend recomputing the familiarity measures using the Yonelinas & Jacoby (1995; Journal of Memory and Language) independent remember/know correction to provide estimates of recollection and familiarity. I computed these measures using the mean data reported in the paper, and it seems as though self-referential encoding increases both recollection and familiarity relative to semantic encoding. The corrected familiarity measure using the Yonelinas & Jacoby formulas was .207 and .148 for self-referential and semantic encoding, respectively. However, whether this holds in the individual data remains to be seen. I do believe addressing this is something that will add to the literature and increase the potential impact of this paper.

Author Response: We thank the reviewer for noting the strengths of the study and bringing up this important point. We have re-calculated the familiarity measure with the IRK procedure. Self-encoding resulted in significantly higher memory accuracy for R trials but not for K trial. The rate of K responses was slightly higher but not significant for self-referential encoding compare to semantic encoding (p =.051). We think this is an interesting addition to the existing literature on SRE in K responses.

2) I was unclear as to how source memory was calculated. It seems to be calculated based on the number of trials with the correct source judgment divided by the total number of old trials (separately the self-referential and semantic types). That is, for example, for the ‘Familiar’ source accuracy in the self-referential encoding task, source memory for the background scene was determined as the number of trials receiving an accurate ‘Familiar’ and source memory response divided by the number of old items in self-referential encoding. This is the only way the numbers shown in Table 1 and the Figures make sense, given they are quite low (which would be below chance). Source memory should not be calculated in this way because it confounds item memory with source memory. The most appropriate way to compute these source accuracy measures is to use the number of correctly identified items (e.g., all ‘familiar’ hits) as the denominator and the numerator as the number of correctly identified items labeled familiar that also attracted an old response. Otherwise, it cannot be easily discerned if the source memory results are just a reflection of the pattern of results observed in the item memory measure.

Author Response: Source memory success rates have been recalculated as the reviewer suggested, with the denominator being the total number of relevant hits (R or K) for the self and semantic encoding conditions. Results are included in Table 2. We focused on source accuracy in R trials only, given that source judgements after a “K” response were most likely to be guesses. The updated results suggest that source accuracy was higher for the self encoding condition compared to semantic condition for recollection memory. The semantic condition resulted in significantly higher proportion of item-only (incorrect source) trials.

Minor Points:
3) The data reported in Table 1 is not entirely clear. The Remembered I data is not the raw proportion of remember responses but is instead the Hits – FA for R responses. This should be made clear, as well as the other columns. I would also recommend including a table of the total proportions of each cell formed by crossing RKN response with source accuracy (e.g., both correct, encoding task only, background scene only). This will help readers be able to make additional comparisons in the group data and will be helpful in identifying potential response bias effects (i.e., were false alarms more likely to be followed by a self-referential task judgment relative to a semantic task judgment). In fact, it would be helpful to report source misattributions.

Author Response: We have restructured Table 1 to show the raw proportions of the responses for studied and unstudied trials, separately for self and semantic encoding conditions. We also included results on t-tests of the false alarms which suggest self- and semantic- encoding task judgements were equally likely to be selected for false alarms (page 12).

4) The y-axis on the figures can use a line with tick marks. It was difficult to determine the values. Also, the y-axis label ‘% correct’ is a little misleading. Hits – FA for R and F are not % correct, but instead are accuracy or discriminability measures.

Author Response: We have re-created the figures to have the y-axis tick marks and remove the %correct label.

5) The presentation of the source memory results was confusing. The post-hoc comparisons for the background and encoding task measures were reported in the ‘Both Source Correct’ section. These should be moved to their respective sections to facilitate ease of reading.

Author Response: These comparisons were moved to their respective sections in order to remedy the confusion.

6) What version of the RF instructions were used? There is a lot of variability in this task, and including the instructions in the OSF repository (or as an appendix in the paper) will help readers understand how the task was done and allow for better replication of the method.

Author Response: We have included a copy of the instructions in the supplementary material. The RK instructions followed closely to Gardiner 1998, Memory and Cognition. Participants were instructed to make the “Remember” judgement if they remember seeing the object during the study session and can recall additional information of the episode such as what they were thinking at the time, and make the “Familiar” judgement if they think they have seen the object in the study phase but were unable to remember anything specific. All participants were given a practice after the instruction, before beginning the task.

Suggestion:
7) My last comment is not something that needs to be addressed unless the authors wish to pursue it. It is simply a suggestion based on a thought I had while reading the paper. As I noted above, a strength of this paradigm is the inclusion of multiple source details. As the authors noted, self-referential encoding may lead to the formation of richer memory traces that support subsequent recollection, as evident by the reported increase in the proportion of trials where both sources was remembered. One way to formally test this would be to use multinomial processing tree model of the RKN and source memory data similar to Boywitt et al. (2012; Psychology and Aging). This approach allows for estimation of a parameter that reflects the degree to which multiple source detail are recollected bound or independent of one another (separately for R and F trials). I think this would be interesting and would help get around the issue I mentioned above regarding estimation of source accuracy measures and potentially provide an informative insight into how self-referential encoding supports recollection (i.e., by increasing binding amongst multiple elements, both central and peripheral, to an event).

Author Response: We now include the mutinomial tree model in the manuscript and supplemental information to test for the probability of retrieving one source when the other source has been retrieved. Two models are made, switching the location of both source types in order to get the results for each source given the success of the other source type. Details of the models (with 9 branches) is included in the supplementary information with the mpt data files in the OSF repository. Our analysis of the MPT results found little significant influence on the successful recollection of source information given the success/failure to recall the other piece of source information. The model also confirmed that there were no biases in the False Alarm answering patterns of participants.
